# Immunotherapy combined with apatinib in the treatment of advanced or metastatic gastric/gastroesophageal tumors: a systematic review and meta-analysis

**DOI:** 10.1186/s12885-024-12340-4

**Published:** 2024-05-17

**Authors:** Jincheng Wang, Jie Lin, Ruimin Wang, Ti Tong, Yinghao Zhao

**Affiliations:** 1 Department of Thoracic Surgery, the Second Hospital of Jilin University, Changchun City, China; 2 Department of Hepatobiliary and Pancreatic Surgery, the Second Hospital of Jilin University, Changchun City, 130000 Jilin China; 3 Department of Operating Room, The Second Hospital of Jilin University, Changchun City, 130041 Jilin China

**Keywords:** Immunotherapy, Apatinib, Gastric/gastroesophageal junction tumor, Efficacy, Safety

## Abstract

**Background:**

Immunotherapy or apatinib alone has been used as third-line adjuvant therapy for advanced or metastatic gastric/gastroesophageal junction (G/GEJ) tumors, but the efficacy of combining them with each other for the treatment of patients with advanced or metastatic G/GEJ is unknown; therefore, we further evaluated the efficacy and safety of immunotherapy combined with apatinib in patients with advanced or metastatic G/GEJ.

**Methods:**

The main search was conducted on published databases: Embase, Cochrane library, PubMed.The search was conducted from the establishment of the database to December 2023.Clinical trials with patients with advanced or metastatic G/GEJ and immunotherapy combined with apatinib as the study variable were collected. Review Manager 5.4 software as well as stata 15.0 software were used for meta-analysis.

**Results:**

A total of 651 patients from 19 articles were included in this meta-analysis. In the included studies, immunotherapy combined with apatinib had a complete response (CR) of 0.03 (95% CI: 0.00 -0.06), partial response (PR) of 0.34 (95% CI: 0.19–0.49), stable disease (SD) of 0.43 (95% CI: 0.32–0.55), objective response rate (ORR) was 0.36 (95% CI: 0.23–0.48), disease control rate (DCR) was 0.80 (95% CI: 0.74–0.86), and median progression-free survival (PFS) was 4.29 (95% CI: 4.05–4.52), median Overall survival (OS) was 8.79 (95% CI: 7.92–9.66), and the incidence of grade ≥ 3 TRAEs was 0.34 (95% CI: 0:19-0.49). PR, ORR, DCR, median PFS and median OS were significantly higher in the immunotherapy and apatinib combination chemotherapy group (IAC) than in the immunotherapy combination apatinib group (IA). And the difference was not significant in the incidence of SD and grade ≥ 3 TRAEs.

**Conclusion:**

This meta-analysis shows that immunotherapy combined with apatinib is safe and effective in the treatment of advanced or metastatic G/GEJ, where IAC can be a recommended adjuvant treatment option for patients with advanced or metastatic G/GEJ. However, more large multicenter randomized studies are urgently needed to reveal the long-term outcomes of immunotherapy combined with apatinib treatment.

**Supplementary Information:**

The online version contains supplementary material available at 10.1186/s12885-024-12340-4.

## Introduction

Gastric or gastroesophageal junction (G/GEJ) tumors are a common malignancy with a rather poor prognosis, which ranks as the fifth most common malignancy and the third leading cause of cancer deaths, and the majority of cases are diagnosed at an advanced stage [1, 2]. With respect to advanced or metastatic G/GEJ adenocarcinomas that do not express HER2, fluoropyrimidine plus platinum-based systemic chemotherapy regimens remain the mainstay of first-line treatment [3]. The second-line treatment of advanced gastric cancer with paclitaxel, irinotecan, doxorubicin, or combination paclitaxel. The Chinese Society of Clinical Oncology (CSCO) guidelines for third-line treatment recommend the use of apatinib, nivulizumab, pembrolizumab, or a rational choice of chemotherapy regimen with reference to the second-line recommended modality [4]. Nevertheless, there are study data suggesting objective remission rates (ORR) of 6.8–25% and progression-free survival (PFS) of 1.5–5.3 months in second or second-line therapy [5–7]. At the same time, with the development of the patient’s condition, the drug resistance of conventional chemotherapy drugs gradually increased, and the clinical application effect decreased significantly [8]. There is an urgent need to develop more effective therapeutic options for the follow-up of patients with advanced or metastatic G/GEJ.

With the emergence of checkpoint inhibitors has led to fundamental changes in the treatment of a number of tumors. Anti-programmed death-1 (anti -programmed death-1,PD-1) antibodies and their ligand, PD-L1 antibodies, have shown antitumor efficacy in a variety of cancers, of which, pembrolizumab has been approved as a third-line treatment for PD-L1-expressing advanced GC [9]. On the other hand, only about 10% of patients with advanced GC/ GEJ benefit from monotherapy [10, 11]. A number of studies have revealed that combining immunotherapy with other treatments could produce a substantial impact on patients with advanced cancer [12–14]. There has been much interest in recent years in the efficacy of anti-pd -1 combined with molecular antiangiogenic drugs. Antiangiogenesis is an established tumor microenvironment (TME)-targeted therapy for GC/GEJ. It may be possible to overcome primary resistance in patients with advanced GC/GEJ by combining PD-1/PD-L1 blockade with agents capable of eliminating pre-existing immunosuppression in the TME [15–17]. Recent studies of the selective VEGFR1-3 inhibitor axitinib in combination with pembrolizumab for the treatment of patients with advanced renal cell carcinoma have reported promising antitumor activity and an acceptable safety profile [18]. Another study of the combination of an anti-pd - l1 antibody (atezolizumab) and a vegf antibody (bevacizumab) also showed encouraging response rates in patients with advanced HCC who tolerated the toxicity [19].

Apatinib is a selective VEGFR2 TKI approved for the treatment of advanced gastric cancer in China [20]. A potential additive or synergistic anti-tumor effect between anti-pd-1 antibodies and VEGF/VEGFR2 inhibitors as demonstrated in vitro and phase I clinical studies [21, 22]. The aim of this meta-analysis is to demonstrate the efficacy and safety of immunotherapy in combination with apatinib in the treatment of advanced or metastatic G/GEJ based on the available data, and to provide further therapeutic options for better survival benefit in advanced or metastatic G/GEJ in the future. Until now, there is no published meta-analysis on a similar topic.

## Methods

### Data sources and search strategy

We conducted an independent systematic literature search mainly in PubMed, Embase, Cochrane Library and Web of Science databases. Recent unpublished clinical trials of immunotherapy combined with apatinib for advanced or metastatic G/GEJ tumors from the American Society of Clinical Oncology (ASCO), the European Society for Medical Oncology (ESMO), and other international oncology congresses were included. The time span was from the inception of the database to December 1, 2023. All keywords were searched by MeSH, mainly including “immunologic agents”, “apatinib”, “advanced or metastatic”, " gastric cancer” and “gastroesophageal junction tumor”. This systematic evaluation and meta-analysis followed the Preferred Reporting Items for Systematic Evaluation and Meta-Analysis (PRISMA) statement [23]. The systematic evaluation and meta-analysis is registered with PROSPERO (registration number: CRD42023491167).

### Inclusion and exclusion criteria

In this meta-analysis, the inclusion criteria were as follows: (1) patients with histopathologically confirmed advanced or metastatic G/GEJ; (2) immunotherapy combined with apatinib as the primary therapeutic agent; and (3) reported at least one of the following primary outcomes: incidence of CR, PR, SD, ORR, DCR, median PFS, median OS, and ≥ 3TRAEs. Exclusion criteria were as follows: (1) patients with resectable or locally advanced G/GEJ; (2) case reports, reviews, or commentaries; (3) multiple articles published by different authors with overlapping or duplicated data; (4) articles not in English; and (5) studies that did not address the key findings of the current meta-analysis.

### Data extraction and quality assessment

Two authors (JCW and JL) independently filtered the titles and abstracts of all included studies. The abstracts of all potentially eligible trials were read independently by the same authors who decided whether the study was selected. The full text of all selected papers was then analyzed by the same author to select all trials that were ultimately included in the combined analysis. When discrepancies in trial search or selection arose, they were discussed with a third researcher (RMW) to reach a final consensus. Data were recorded and archived in an Excel spreadsheet. In addition, parameters were extracted in a uniform format, including first author, year of publication, study type (single-arm or RCT), approval number, dMMR/pMMR, HER2, PD-L1 expression, pathologic typing, pathologic staging, treatment modality, number of enrollees, age, incidence of ≥ grade 3 TRAEs, CR, PR, ORR, SD, DCR, median PFS, and median OS. The partial MINORS tool was used to evaluate the study quality. The items are scored 0 (not reported), 1 (reported but inadequate), or 2 (reported and adequate) [24].

### Statistical analysis

Meta-analysis of non-comparative binary outcomes was mainly applied because most of the included studies were single-arm clinical studies and the outcome indicators were mainly expressed as proportions. The combined odds ratios (OR) and 95% confidence intervals (CI) were converted into incidence rates to assess the efficacy and safety of immunotherapy in combination with apatinib in the treatment of advanced/metastatic G/GEJ. q-tests of *P* < 0.05 or I2 > 50% were used to consider that there was significant heterogeneity in the literature, and a random-effects model was used; otherwise, a fixed-effects model was applied. In addition, sensitivity analyses were performed by sequentially removing individual studies to assess the stability of the combined results of these studies. For studies with significant heterogeneity that could not be reduced using sensitivity analyses, further subgroup analyses were performed to explore the sources of heterogeneity. A funnel plot test for publication bias was used. *p* < 0.05 was considered a statistically significant difference. In addition, as median PFS and median OS were continuous variables that could not be calculated using incidence for analysis, further analysis using stata 15.0 software was required. All analyses were performed using Review Manager 5.4/stata 15.0 software.

## Results

### The characteristics of the included studies

**Fig. 1:**
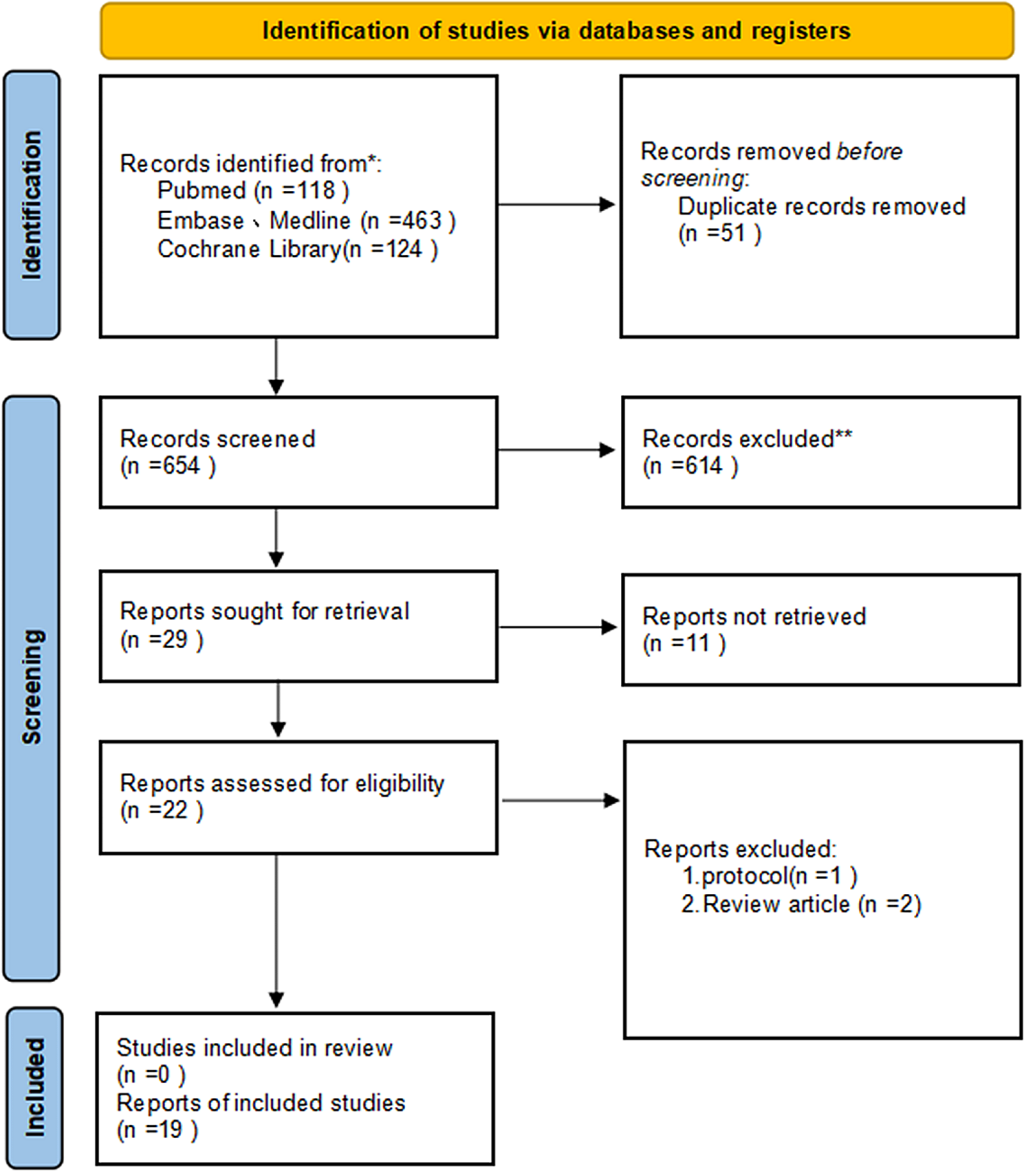
Preferred Reporting Items for Systematic Reviews and Meta-Analyses (PRISMA) diagram of the study selection

There is a PRISMA diagram of the study selection process as shown in Fig. 1. According to the search strategy, a total of 705 publications were included (118 PubMed, 463 Embase, and 124 Web of science), and 19 studies [21, 25–42] with a total of 651 patients were eligible for inclusion in the final meta-analysis. meta-analysis included a total of 16 single-arm cohort studies, and three randomized controlled studies. The main immunotherapeutic agents were SHR-1210, JS001, Camrelizumab, Pembrolizumab, sintilmab, Tislelizumab, and Nivolumab.Depending on the treatment regimen, we can categorize them into 2 main therapeutic modalities, namely, immunotherapy combined with apatinib (IA) and immunotherapy and apatinib combined with chemotherapy (IAC). The main characteristics of the included studies are shown in Table 1, and the main outcomes are shown in Table 2. Supplementary Table 1 shows the overall low risk of bias of the included studies.

**Table 1:** Main characteristics of included studies

Author year	NCT number	Study design	TYPE	PD−1/ PD-L1	Combination therapy	Clinical stage	No. of patient	Median age	Tumor type	Preoperative PD-L1 CPS	dMMR/pMMR	Her2
Jianming Xu2018	NCT03463876	IA	Single-arm	SHR−1210	Apatinib	Advanced/Metastatic	25	54	G/GEJ	-	-	-
Qing Wei2020	-	IA	Single-arm	SHR−1210/JS001/N/S	Apatinib	Advanced	24	60.5	G/GEJ	-	-	-
Li-Hua Li2022	-	IA	Single-arm	C/S/N	Apatinib	Advanced	39	61	G/GEJ	-	-	Positive 1 Negative 17 Unknown 21
Ning Ma2022	-	IA	Single-arm	C	Apatinib	Metastatic	19	63	G	< 50% 8 50% 11	-	-
Loulu Gao2023	-	IA	Single-arm	S	Apatinib	Advanced/Metastatic	34	61	G/GEJ	CPS < 1 5 CPS ≥ 1 2 Unknown 27	pMMR 12 dMMR 2 Unknown 20	Positive 4 Negative 20 Unknown 10
L. Xiao2020	NCT04067986	IA	Single-arm	C	Apatinib	Metastatic	15	-	G	-	-	-
Beibei Chen2022	-	IA	Single-arm	PD−1	Apatinib	Advanced	34	-	G	-	-	-
Hou X.-F2023	-	IA	Single-arm	T/N	Apatinib	Advanced/Metastatic	52	-	G/GEJ	-	-	Negative
Qingli Cui2022	-	IA	RCT	PD−1	Apatinib	Advanced	49	63	G	CPS < 1 28 CPS ≥ 1 20 Unknown 1	pMMR 49 dMMR 0 Unknown 0	Positive 9 Negetive 40
Caiyun Nie2022	-	IA	RCT	PD−1	Apatinib	Advanced/Metastatic	54	59	G	-	-	Positive 7 Negative 47
Miaomiao Gou2023	-	IA	RCT	P/N/S/C/T	Apatinib	Metastatic	51	-	G	-	-	-
Zhi Peng2021	NCT03472365	IAC	Single-arm	C	Apatinib + CAPOX	Advanced/Metastatic	48	56	G/GEJ	CPS > 1 15 CPS ≤ 1 13 Unknown 20	-	-
Chao Jing2022	NCT04345783	IAC	Single-arm	C	Apatinib + S−1	Advanced/Metastatic	24	64	G/GEJ	CPS ≥ 10 3 CPS ≥ 1 9 CPS < 1 10	pMMR 17 dMMR 2	Negative
Kunpeng Wu2023	-	IAC	Single-arm	C	Apatinib + TACE	Advanced	49	67	G/GEJ	-	-	-
Le Zhang2023	NCT05025033	IAC	Single-arm	S	Apatinib + chemotherapy	Advanced	30	59	G/GEJ	-	pMMR 30	-
Ting Deng2021	-	IAC	Single-arm	S	Apatinib + chemotherapy	Advanced	26	61	G/GEJ	-	-	-
Miaomiao Gou2022	NCT04182724	IAC	Single-arm	PD−1	Apatinib + Albumin paclitaxel	Metastatic	23	-	G	-	-	-
L. Su2022	NCT04174339	IAC	Single-arm	C	Apatinib + POF	Advanced	20	-	G	-	-	Negative
Xiaofeng Chen2023	-	IAC	Single-arm	C	Apatinib + SOX	Advanced	35	59	G/GEJ	-	-	-

IA: Immunotherapy combined with apatinib ; IAC: Immunotherapy and apatinib combined with chemotherapy ; C: Camrelizumab ; P: Pembrolizumab ; S: Sintilimab ; T: Tislelizumab ; N: Nivolumab ; CAPOX: capecitabine + oxaliplatin ; SOX: S-1 + Oxaliplatin ; TACE: Transcatheter arterial chemoembolization ; POF: Paclitaxel + oxaliplatin + L-folate + 5-FU; CPS: Combined Positive Score ; dMMR: Mismatch Repair Deficiency; pMMR: Proficient mismatch repair

**Table 2:** Main characteristics of included studies

Author year	CR	PR	ORR	SD	PD	Not evaluable	DCR	≥ 3 TRAEs	Median PFS (month)	Median OS (month)
Jianming Xu2018	-	4/23	4/23	13/23	5/23	2/25	17/23	-	2.9(95%CI 2.5–4.2)	11.4(95%CI 8.6-NR)
Qing Wei2020	1/19	4/19	5/19	7/19	7/19	5/24	12/19	3/19	3.0(95%CI 1.3–4.7)	-
Li-Hua Li2022	-	8/39	8/39	19/39	12/39	-	27/39	21/39	3.9(95%CI 2.74–5.06)	7.8(95%CI 4.82–10.78)
Ning Ma2022	-	5/19	5/19	8/19	6/19	-	13/19	-	7.0(95%CI 2.9–11)	10.0(95%CI 7.4–12.6)
Loulu Gao023	-	3/34	3/34	23/34	8/34	-	26/34	-	6.0(95%CI 3.6–8.4)	11.6(95%CI 8.1–15.1)
L. Xiao2020	-	3/15	3/15	8/15	4/15	-	11/15	-	-	-
Beibei Chen2022	-	2/34	2/34	17/34	15/34	-	19/34	-	2.47(95%CI 1.9−3.0)	6.8(95%CI 3.7–9.9)
Hou X.-F2023	-	-	8/52	24/52	20/52	-	32/52	12/52	4.2(95%CI 2.6–4.8)	9.3(95%CI 7.9–12.9)
Qingli Cui2022	-	-	17/49	20/49	12/49	-	37/49	17/49	5.5(95%CI 3.7–7.3)	10.0(95%CI 5.3–13.7)
Caiyun Nie2022	-	10/54	10/54	24/54	20/54	-	34/54	10/54	3.0(95%CI 2.4–3.6)	5.2(95%CI 3.4−7.0)
Miaomiao Gou2023	-	-	10/51	38/51	3/51	-	48/51	10/48	4.1(95%CI 3.51–4.68)	7.6(95%CI 5.34–9.85)
Zhi Peng2021	1/46	27/46	28/46	17/46	1/46	2/48	45/46	6/24	6.8(95%CI 5.6–9.5)	14.9(95%CI 13.0−18.6)
Chao Jing2022	1/24	6/24	7/24	16/24	3/24	-	23/24	5/49	6.5(95%CI 6.01–6.99)	-
Kunpeng Wu2023	2/49	28/49	30/49	18/49	1/49	-	48/49	-	-	20.0(95%CI 13.6–26.4)
Le Zhang2023	-	15/28	15/28	8/28	5/28	2/30	23/28	-	8.5(95%CI 5.4–11.5)	12.5(95%CI 3.7–21.3)
Ting Deng2021	-	12/24	12/24	8/24	4/24	2/26	20/24	-	7.06(95%CI 5.52–8.60)	-
Miaomiao Gou2022	-	8/23	8/23	10/23	5/23	-	18/23	18/20	5.04	-
L. Su2022	-	-	16/20	4/20	0	-	20/20	16/35	11.0(95%CI 7.0–15.0)	14
Xiaofeng Chen2023	-	30/33	30/33	1/33	2/33	2/35	31/33	10/48	10.2(95%CI 5.5–22.3)	-

CR: Complete response ; PR: Partial response ; SD: Stable disease ; ORR: Objective response rate ; DCR: Disease control rate ; TRAEs: Treatment-related adverse event ; PD: Progressive disease; PFS: Progression-Free Survival ; OS: Overall Survival

### Evaluation of efficacy outcomes

**Fig. 2:**
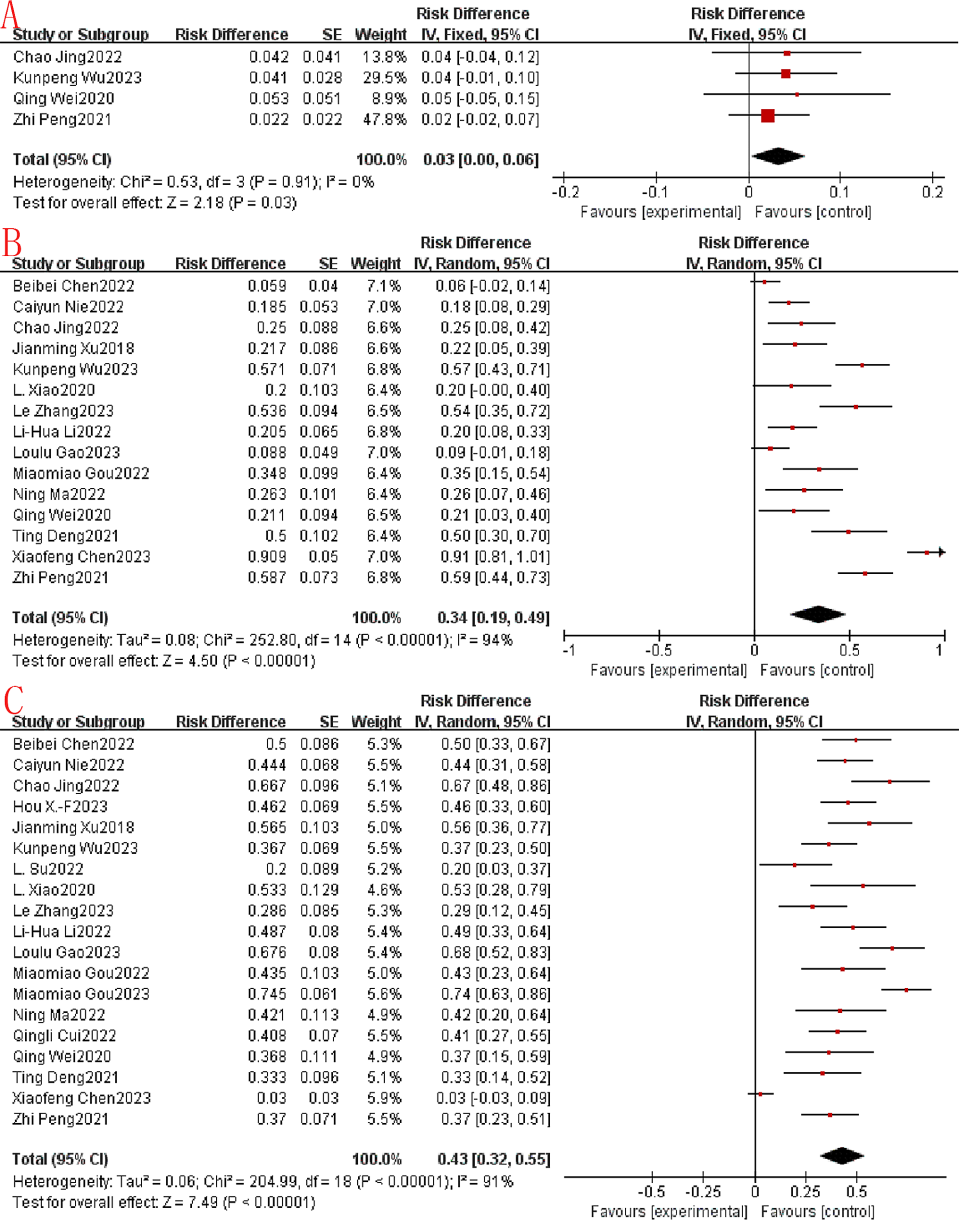
Immunotherapy combined with apatinib forest plot. (**A**): CR; (**B**): PR. (**C**): SD

In this study, CR, PR, SD, ORR and DCR were used to evaluate the efficacy of immunoapatinib treatment. CR is when a tumor has been treated so that all previously detectable tumors have disappeared and there is no clinical or imaging evidence of tumor presence. Of all the included studies, CR was not assessed in 4 studies, CR was not achieved in 11 studies, and CR in the remaining studies ranged from 2.2 to 5.3%. In the four eligible studies, the combined CR was 0.03 (95% CI: 0.00 -0.06), a statistically significant difference (*p* = 0.03). Using a fixed-effects model, there was no significant heterogeneity among the 4 studies (*P* = 0.91, I^2^ = 0%; Fig. 2A). PR was defined as a ≥ 30% reduction in the sum of the largest diameters of the tumor target lesions, maintained for at least 4 weeks. Meanwhile, among the 15 eligible studies, the combined PR was 0.34 (95% CI: 0.19–0.49), a statistically significant difference (*P* < 0.00001). Using a random-effects model, there was significant heterogeneity among the 15 studies (*p* < 0.00001, I^2^ = 94%; Fig. 2B). SD is defined as shrinkage of the sum of the largest diameters of the tumor target lesions without PR, or enlargement without disease progression. Among the 19 eligible studies, the combined SD was 0.43 (95% CI: 0.32–0.55), a statistically significant difference (*p* < 0.00001). Using a random-effects model, there was similarly significant heterogeneity among the 15 studies (*p* < 0.00001, I^2^ = 91%; Fig. 2C).

**Fig. 3:**
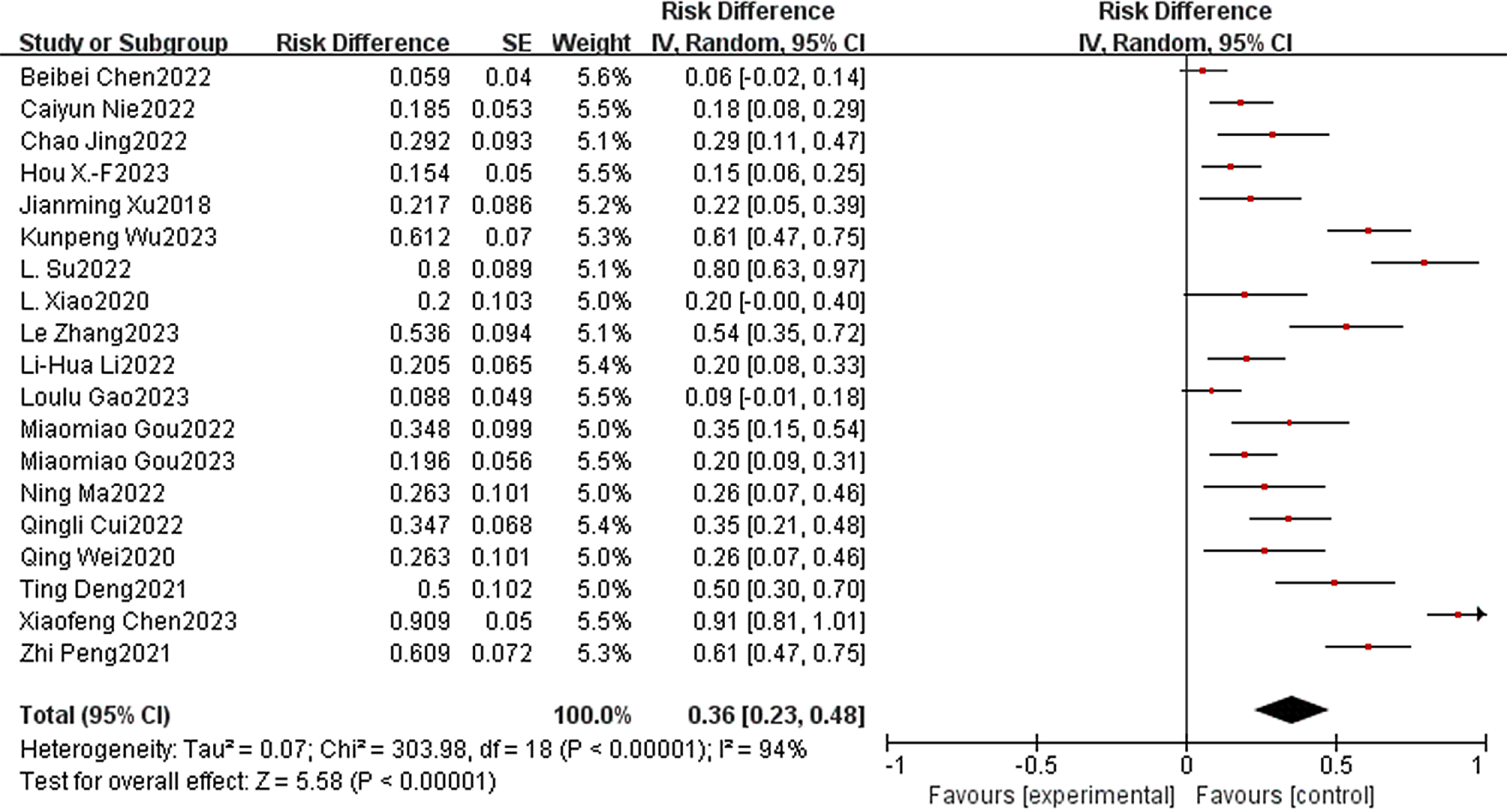
Immunotherapy combined with apatinib forest plot.:(ORR)

**Fig. 4:**
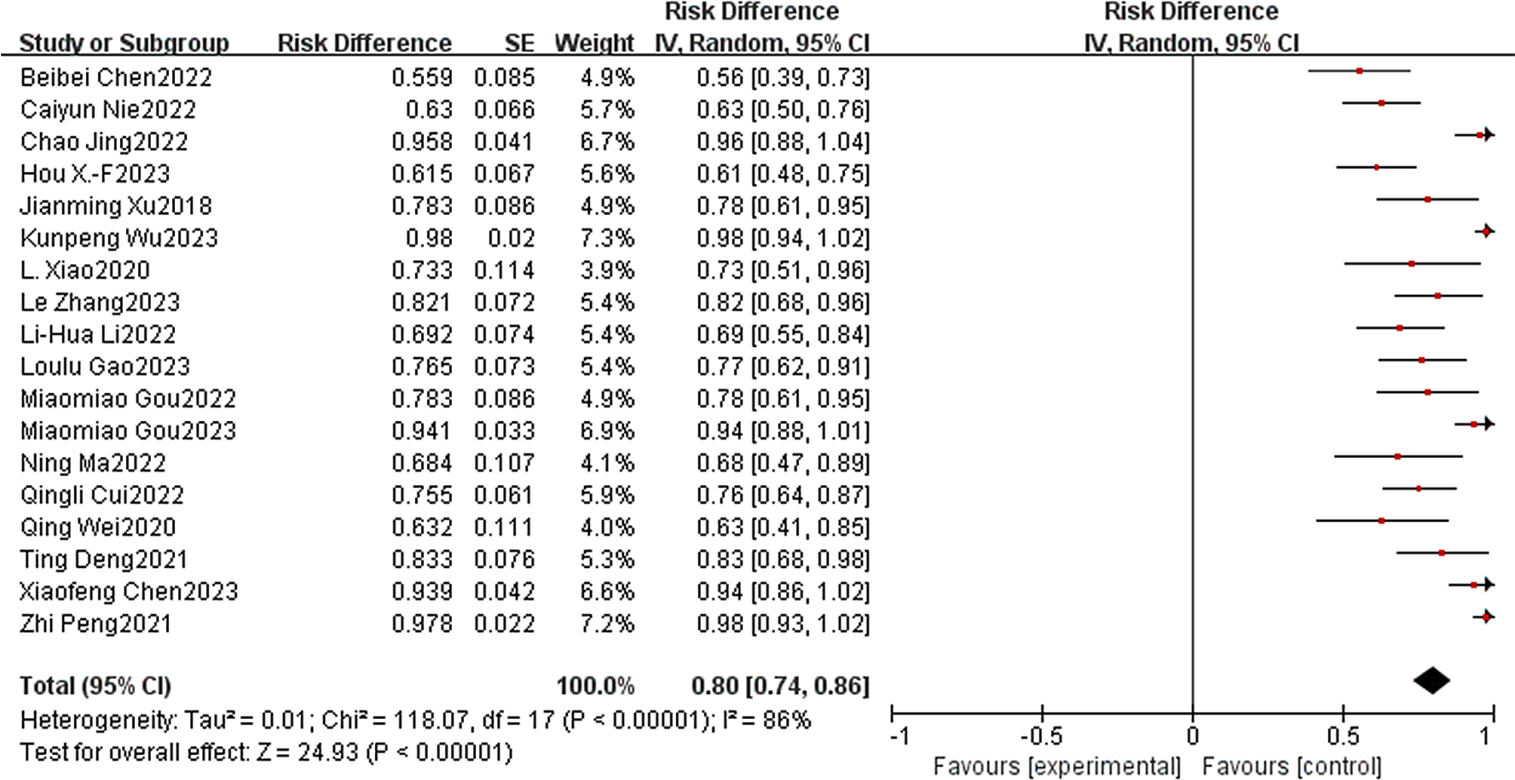
Immunotherapy combined with apatinib forest plot.:(DCR)

ORR is the proportion of patients whose tumor volume shrinks to a pre-specified value and maintains the minimum timeframe requirement, and is the sum of the CR and PR proportions. In addition, the 19 included studies reported ORR rates ranging from 5.9 to 90.9%. The joint ORR was 0.36 (95% CI: 0.23–0.48), a statistically significant difference (*p* < 0.0001). Using a random-effects model, there was significant heterogeneity among the 19 studies (*p* < 0.0001, I^2^ = 94%; Fig. 3). DCR is the number of cases that achieved remission (PR + CR) and lesion stabilization (SD) after treatment as a percentage of the number of evaluable cases. In contrast, a total of 18 studies could be included in the DCR for single-arm Meta-analysis, with a joint DCR of 0.80 (95% CI: 0.74–0.86), a statistically significant difference (*p* < 0.0001). Using a random-effects model, there was similarly significant heterogeneity among the 18 studies (*p* < 0.0001, I^2^ = 86%; Fig. 4).

**Fig. 5:**
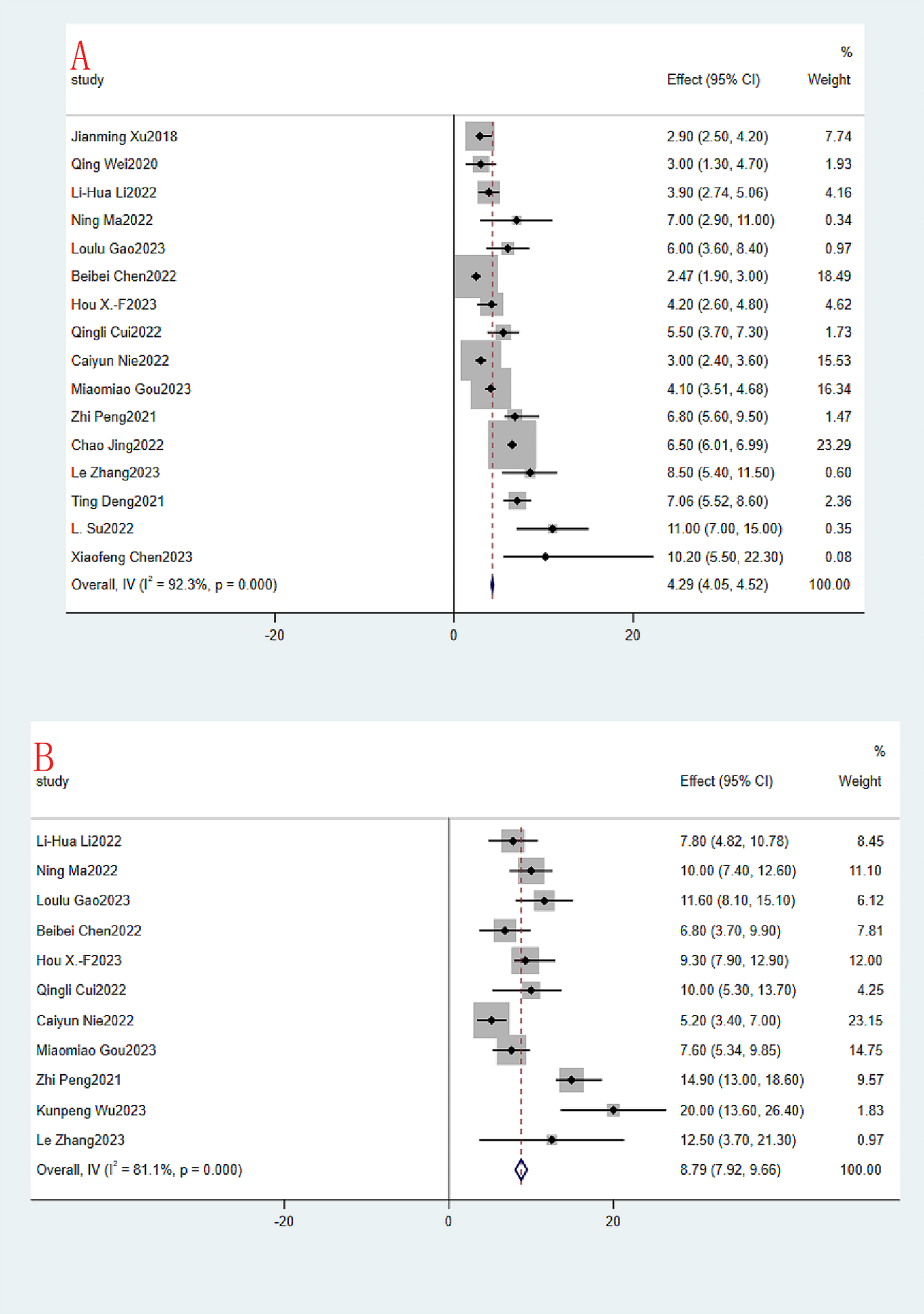
Immunotherapy combined with apatinib forest plot. (**A**): Median PFS; (**B**): Median OS

The same was true for median PFS and median OS, with 16 and 11 studies included, respectively. The median PFS (month) ranged from 2.47 to 11, and the OR of the combined median PFS was 4.29 (95% CI:4.05–4.52, I^2^ = 92.3%,*P* = 0.000, Fig. 5A). Due to the large heterogeneity of the 16 studies, a random effects model was used. The median OS (month) of course also ranged from 5.2 to 20, and the OR of the combined median OS was 8.79 (95% CI:7.92–9.66, I^2^ = 81.1%,*P* = 0.000, Fig. 5B). Again, due to the large heterogeneity of the 11 included studies, a random effects model was used.

### Evaluation of safety outcomes

**Fig. 6:**
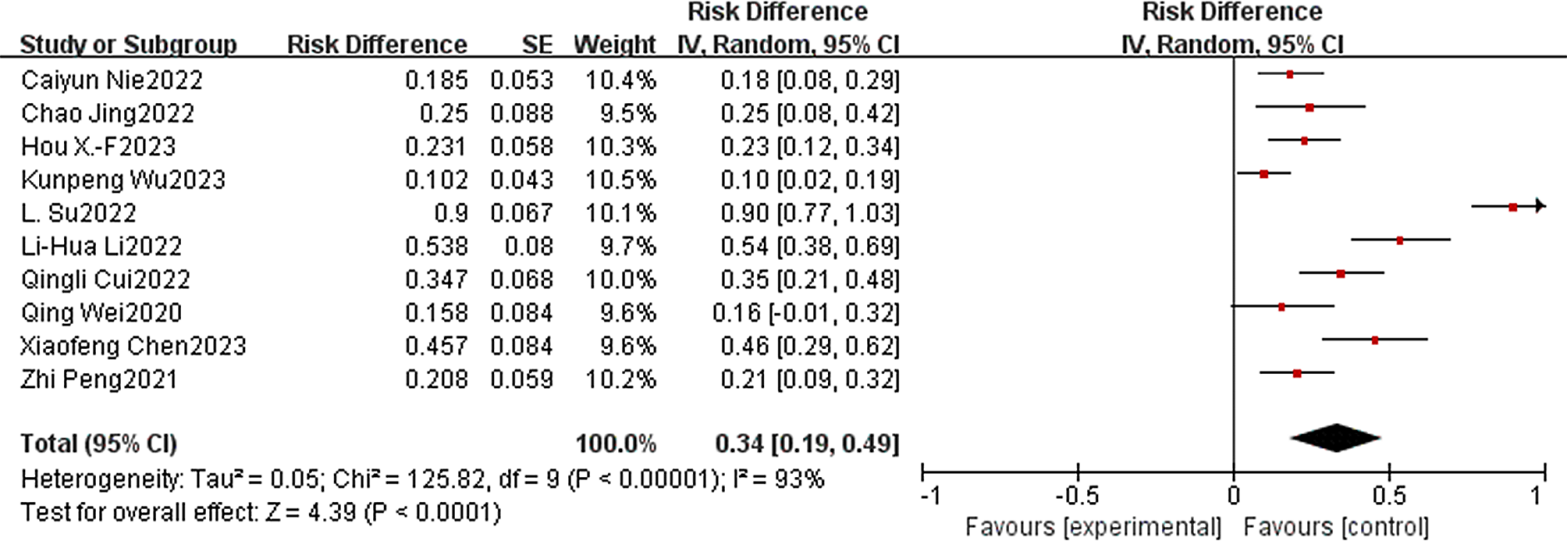
Immunotherapy combined with apatinib forest plot: (≥ 3 TRAEs)

The safety of immunotherapy in combination with apatinib for the treatment of patients with advanced or metastatic G/GEJ was evaluated according to the National Cancer Institute Common Terminology Criteria for Adverse Events (NCI-CTCAE16; version 4.0) [43]. A total of 10 of the included clinical studies reported the incidence of grade ≥ 3 and higher treatments, totaling 118 patients. The incidence of combined grade ≥ 3 TRAEs was 0.34 (95% CI: 0:19-0.49, I^2^ = 93%, *P* < 0.00001) (Fig. 6). Only 1 patient [35] patient died due to grade ≥ 3 TRAEs (abnormal liver function and interstitial lung disease). Other mainly controllable adverse events such as thrombocytopenia, anemia, neutropenia, leukopenia, pruritus, rash, hand-foot syndrome, elevated AST/ALT, fatigue, nausea and vomiting, diarrhea, hypertension, proteinuria, and reactive cutaneous capillary endothelial cell proliferation, among others, did not result in serious adverse outcomes or lead to mortality.

### Sensitivity analysis and subgroup analysis

**Fig. 7:**
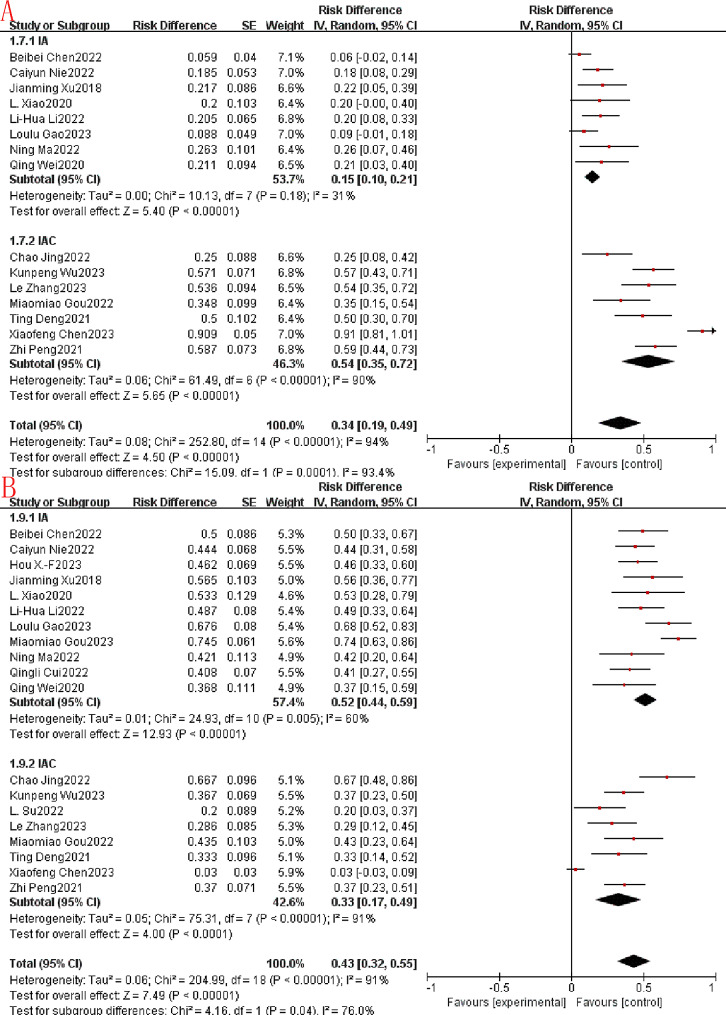
Subgroup analysis. (**A**): PR. (**B**): SD

**Fig. 8:**
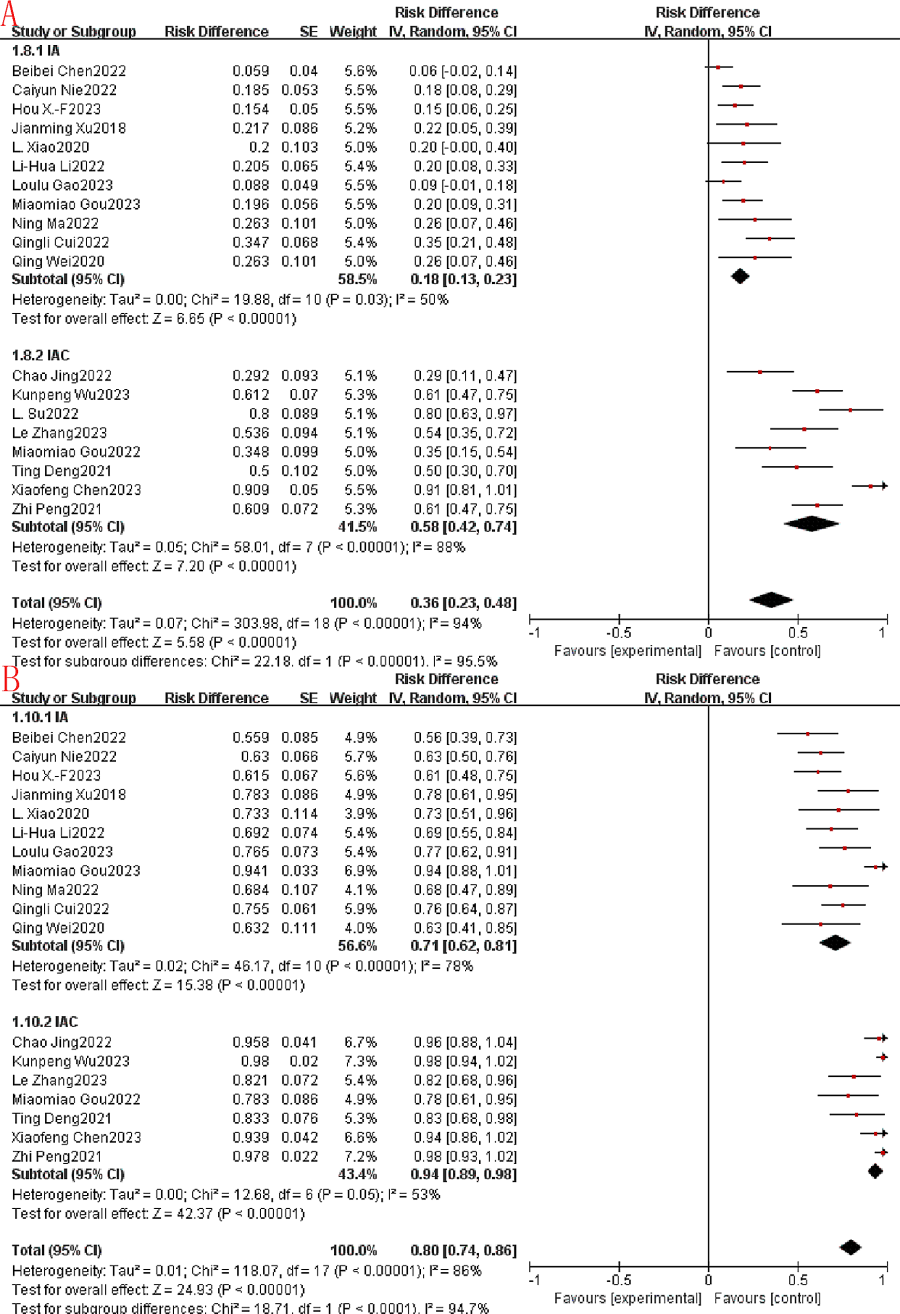
Subgroup analysis. (**A**): ORR. (**B**): DCR

Reconsideration of study search, selection, and inclusion criteria did not reduce heterogeneity. To determine that the joint results were not heavily influenced by individual trials, the included studies were taken out of sequence for sensitivity analysis. We found that this did not significantly reduce heterogeneity. To further identify possible sources of heterogeneity, immunotherapy combined with apatinib was grouped according to whether it was combined with other treatment modalities. In the subgroup analysis, significant differences in PR, SD, ORR, DCR, median PFS and median OS were found. Among them, PR, ORR, DCR, median PFS and median OS were much higher in the IAC group than in the IA group (0.54 vs. 0.15, Fig. 7A.58 vs. 0.18, Fig. 8A.94 vs. 0.71, Fig. 8B.67 vs. 3.35, Fig. 9.85 vs. 15.79, Fig. 10). In terms of the incidence of SD, the IAC group group was slightly lower than the IA group, but the difference was not significant (0.33 vs. 0.52, Fig. 7B). And there was no significant difference between the IAC group and the IA group in terms of grade ≥ 3 TRAEs (0.31 vs. 0.27, I^2^ = 5.8%,*P* = 0.30, Fig. 11). This showed that the IAC group both improved the effectiveness of the treatment without increasing the incidence of adverse events, implying that the combination of immunotherapy and apatinib with chemotherapy may be somehow superior to immunotherapy alone combined with apatinib.

**Fig. 9:**
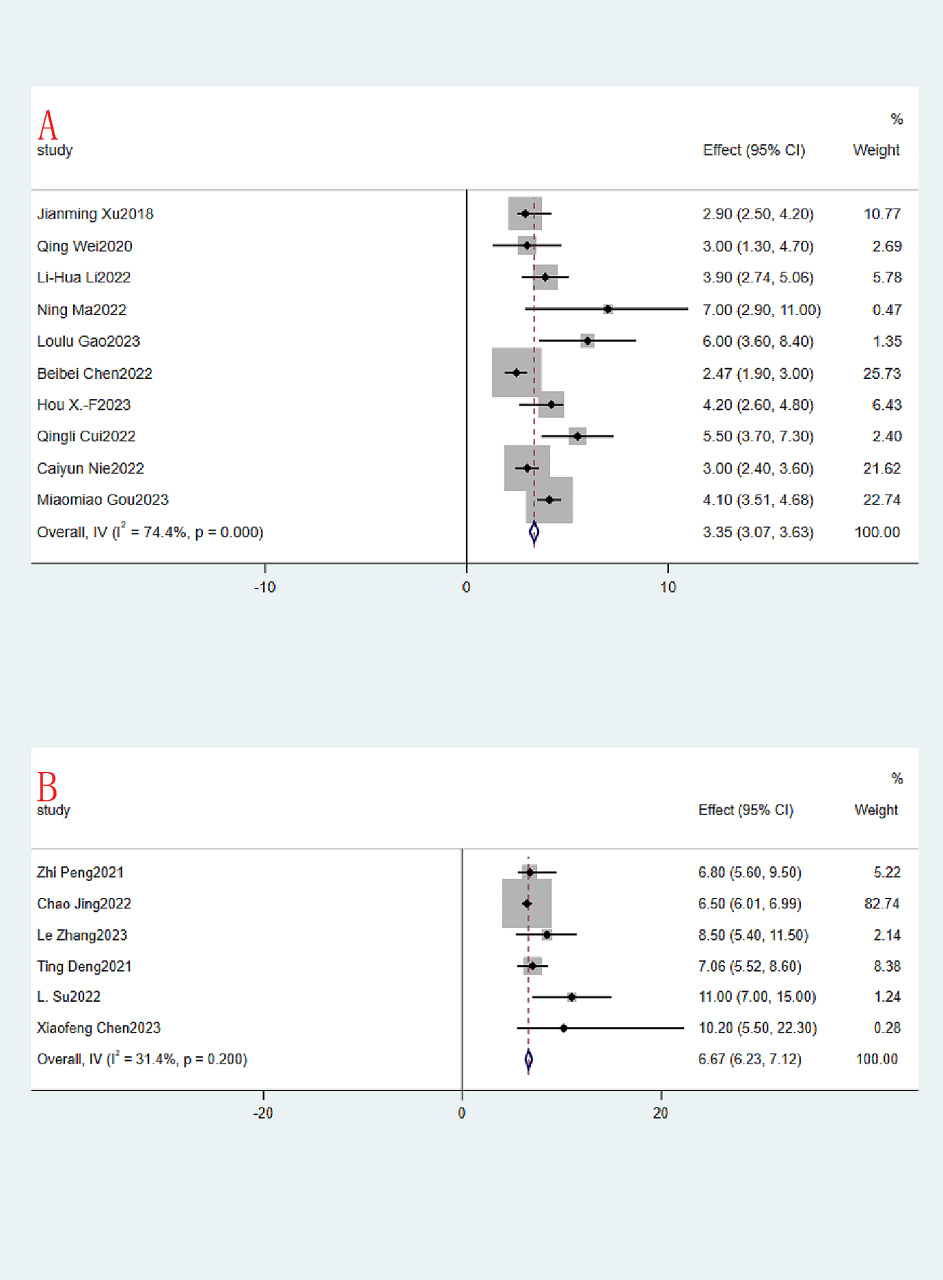
Subgroup analysis of Median PFS. (**A**): IA; (**B**): IAC

**Fig. 10:**
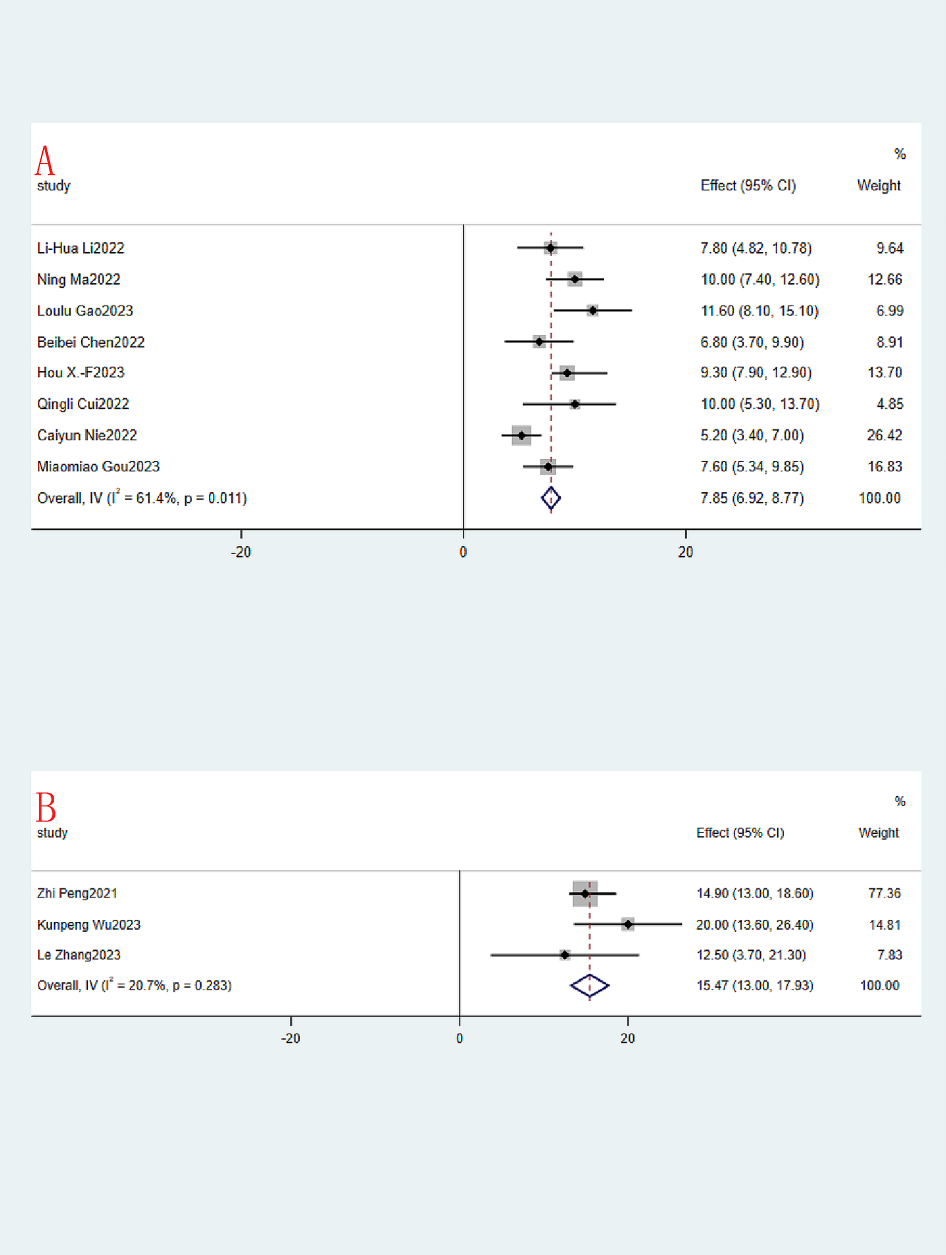
Subgroup analysis of Median OS (**A**): IA; (**B**): IAC

**Fig. 11:**
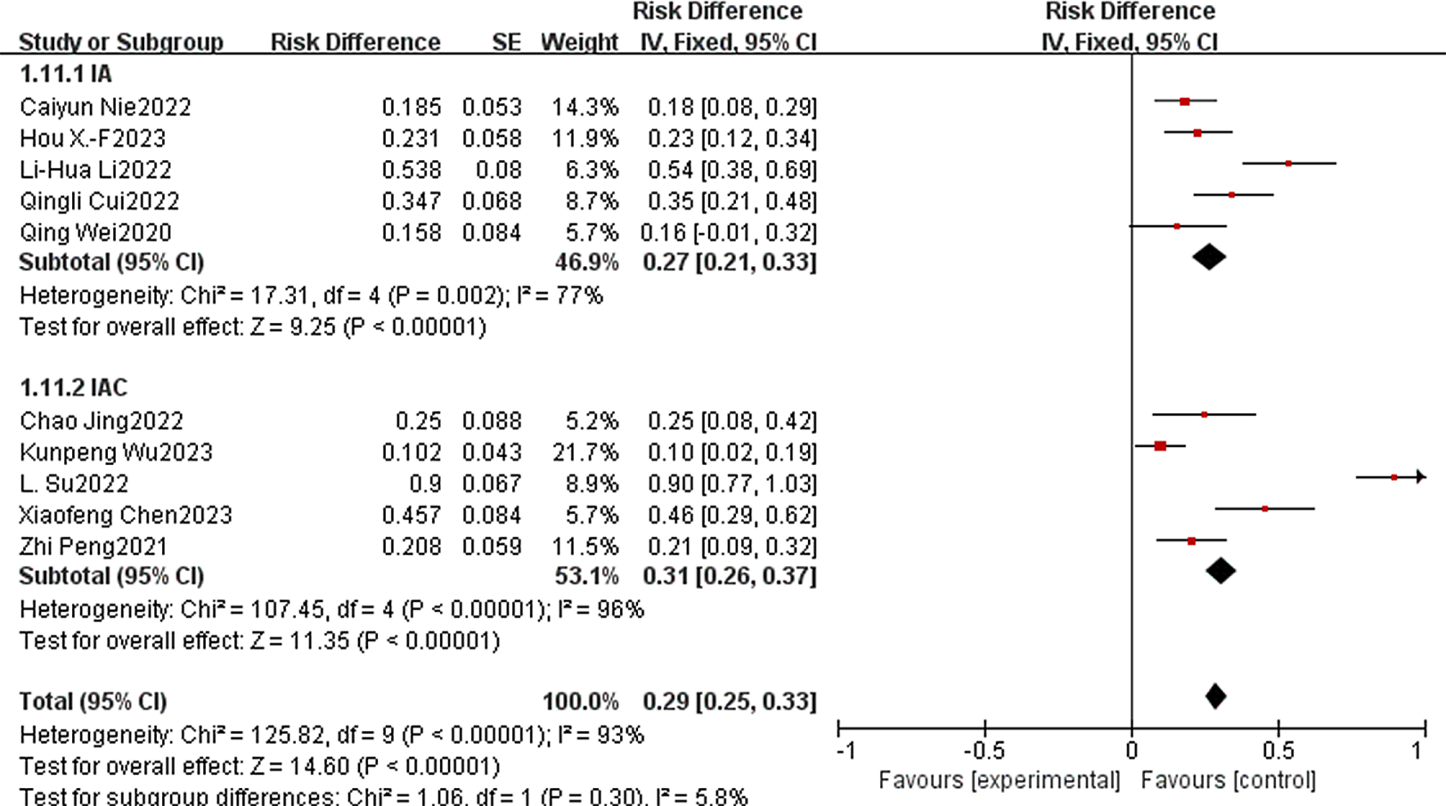
Subgroup analysis of ≥ 3 TRAEs

### Publication bias

Due to the high degree of heterogeneity, most of the above results were obtained using random-effects models, and therefore future phase 3 and large-scale randomized controlled trials are needed for further assessment. Funnel plots were used to analyze possible publication bias for immunotherapy combined with apatinib in 19 clinical studies. Most of the data collected were single-arm clinical trials without controls, but there was no significant publication bias (Supplementary Fig. 1).

## Discussion

Approximately half of the world’s new cases of gastric cancer are detected in China each year, and half of all Chinese patients are diagnosed at an advanced stage. It is recommended in the 2021 CSCO guidelines that fluorouracil in combination with platinum (or) paclitaxel is the first-line standard chemotherapy regimen for patients with her2 negative advanced gastric cancer [4]. But in clinical practice, there are still a large number of patients failing first-line treatment. As a maintenance or sequential treatment chemo-free strategy is constantly being explored. Immunotherapy or apatinib alone has been used in the third-line treatment of advanced or metastatic G/GEJ, but the results remain suboptimal. Regardless of PD-L1 status in the ATTRACTION-2 trial, nivolumab monotherapy improved overall survival in patients with advanced gastric cancer by 5.26 months (95% CI 4.60–6.37), but the ORR was only 11.2% (95% CI: 7.77–15.6) [10]. Based on previous studies, the median PFS and OS of mGC patients receiving third-line treatment with apatinib monotherapy were 2.70 ∼ 4.47 months and 4.27–6.51 months, respectively [20, 44, 45]. Some findings suggest that combination therapy with PD-1 inhibitors and apatinib improves therapeutic efficacy, mainly because tumor angiogenesis inhibits the extravasation of reactive T-cells, which form an immunosuppressive microenvironment, leading to tumor evasion of immune surveillance. Combination therapy enhances t-cell infiltration and activation, thereby eliminating tumor cells [46–49]. A combination study of PD-1 monoclonal antibodies and angiogenesis inhibitors has been initially validated in several clinical trials. Ramucirumab in combination with nivolumab or pembrolizumab has shown promising efficacy in AGC patients in several phase I/II trials [50–52].

As far as we know, this is the first meta-analysis evaluating the efficacy and safety of immunotherapy combined with apatinib for the treatment of patients with advanced or metastatic G/GEJ. Our analysis is based on 19 small studies, including 651 patients, quantitatively and synthetically analyzing the efficacy and safety of immunotherapy combined with apatinib treatment. There is great excitement about the results of the current meta-analysis study. The aggregated CR, PR, SD, ORR and DCR for immunotherapy combined with apatinib were 0.03 (95% CI: 0.00 -0.06), 0.34 (95% CI: 0.19–0.49), 0.43 (95% CI: 0.32–0.55), 0.36 (95% CI: 0.23–0.48), 0.80 (95% CI: 0:74-0.86). And the median PFS and median OS reached 4.29 (95% CI:4.05–4.52), 8.79 (95% CI:7.92–9.66), respectively. Subgroup analysis showed significant differences in PR, ORR, DCR, median PFS, and median OS, with the IAC group being significantly better than the IA group as well as the IAC group being slightly lower than the IA group in terms of SD. The IAC group had an ORR of 0.58 (95% CI: 0.42–0.74, Fig. 8A), a DCR of 0.94 (95% CI: 0.89–0.98, Fig. 8B) a median PFS of 6.67 months (95% CI: 6.23–7.12, Fig. 9B) and a median OS of 15.47 months (95% CI. 13.00-17.93, Fig. 10B), which was higher than the ORR (about 40%), PFS (5.5 months) and OS (11.5 months) of fluorouracil-platinum regimen [53]. This suggests that combination chemotherapy with immunotherapy and apatinib is superior to adjuvant chemotherapy in terms of effectiveness. In the IAC group, the L. Su et al. study achieved a 100% DCR as well as the highest median PFS of 11.0 (95% CI: 7.0–15.0) months [41]. In addition, there was the highest median OS of 20.0 months (95% CI: 13.6–26.4) in the study by Kunpeng Wu et al [37]. And from the 3 RCT trials we included, we found that immunotherapy combined with apatinib treatment was superior to immuno/apatinib alone, apatinib combined with chemotherapy, and immuno combined with chemotherapy in terms of median PFS and median OS [32–34].

Considering the safety of immunotherapy combined with apatinib, the combined OR for the incidence of grade ≥ 3 TRAEs was 0.34 (95% CI: 0:19-0.49), which was not significantly different between the IAC and IA groups (0.31 vs. 0.27). Among the studies we included, only 2 studies explicitly stated that a total of 6 and 12 patients, respectively, discontinued their medication because of TRAE due to immunologic agents or apatinib [26, 36]. Only one patient also died from grade ≥ 3 TRAEs, and other adverse events were manageable. In conclusion, combination therapy with immunotherapy and apatinib has shown encouraging clinical activity in patients with advanced or metastatic G/GEJ, which may improve survival and show tolerable toxicity as second- or third-line therapy.

On the other hand, however, the current meta-analysis still has some limitations. First, the small number of included studies, insufficient sample size, and mostly single-arm clinical trials, the lack of randomized controlled trials, and the single type of study may lead to bias. Therefore, more multicenter, large-sample phase III randomized controlled trials and subsequent meta-analysis are needed to further validate the results of this study. Second, more predictive biomarkers are urgently needed to identify patients who benefit most from immunotherapy combined with apatinib treatment. Despite the heterogeneity, the results suggest that adjuvant therapy based on immunotherapy combined with apatinib is safe and feasible with a favorable improvement in survival, pointing the way to the future development of adjuvant therapy for advanced or metastatic G/GEJ.

## Conclusion

This meta-analysis shows that immunotherapy combined with apatinib is safe and effective in the treatment of advanced or metastatic G/GEJ, where IAC can be a recommended adjuvant treatment option for patients with advanced or metastatic G/GEJ. However, more large multicenter randomized studies are urgently needed to reveal the long-term outcomes of immunotherapy combined with apatinib treatment.

### Electronic supplementary material

Below is the link to the electronic supplementary material.

Supplementary Material 1

Supplementary Material 2

Supplementary Material 3

## Data Availability

The original contributions presented in the study are included in the article/Supplementary Material. Further inquiries can be directed to the corresponding author.
