# Supplementary material for: Immunotherapy combined with apatinib in the treatment of advanced or metastatic gastric/gastroesophageal tumors: a systematic review and meta-analysis
Source: BMC Cancer. 2024 May 17;24:603. doi: 10.1186/s12885-024-12340-4 (PMC11102247; doi:10.1186/s12885-024-12340-4)
Supplement: Supplementary file 1 — Supplementary Material 1 [file 12885_2024_12340_MOESM1_ESM.docx]

**Supplementary Table 1** Assessment of non-randomized controlled trials in the version of MINORS.

| Study | A clearly  stated  aim | Inclusion of  consecutive  patients | Prospective  collection of  data | Endpoint  appropriate  to the study  aim | Unbiased  assessment  of endpoints | Follow-up  period  appropriate  to the major  endpoint | Loss to  follow  up not  exceeding  5% | Prospective  calculation  of the study  size | Total score |
| --- | --- | --- | --- | --- | --- | --- | --- | --- | --- |
| Jianming Xu2018 | 2 | 2 | 2 | 2 | 0 | 2 | 2 | 1 | 13 |
| Qing Wei2020 | 2 | 2 | 2 | 2 | 0 | 2 | 2 | 0 | 12 |
| Li-Hua Li2022 | 2 | 2 | 2 | 2 | 0 | 2 | 2 | 1 | 13 |
| Ning Ma2022 | 2 | 2 | 2 | 2 | 0 | 2 | 2 | 1 | 13 |
| Loulu Gao2023 | 2 | 2 | 2 | 2 | 0 | 2 | 2 | 1 | 13 |
| L. Xiao2020 | 2 | 2 | 2 | 2 | 0 | 0 | 2 | 0 | 10 |
| Beibei Chen2022 | 2 | 2 | 2 | 2 | 0 | 2 | 2 | 1 | 13 |
| Hou X.-F2023 | 2 | 2 | 2 | 2 | 0 | 2 | 2 | 0 | 12 |
| Zhi Peng2021 | 2 | 2 | 2 | 2 | 0 | 2 | 2 | 0 | 12 |
| Chao Jing2022 | 2 | 2 | 2 | 2 | 0 | 1 | 2 | 0 | 11 |
| Kunpeng Wu2023 | 2 | 2 | 2 | 2 | 0 | 1 | 2 | 1 | 12 |
| Le Zhang2023 | 2 | 2 | 2 | 2 | 0 | 2 | 2 | 0 | 12 |
| Ting Deng2021 | 2 | 2 | 2 | 2 | 0 | 1 | 2 | 1 | 12 |
| Miaomiao Gou2022 | 2 | 2 | 2 | 2 | 0 | 1 | 2 | 1 | 12 |
| L. Su 2022 | 2 | 2 | 2 | 2 | 0 | 2 | 2 | 0 | 12 |
| Xiaofeng Chen 2023 | 2 | 2 | 2 | 2 | 0 | 1 | 2 | 1 | 12 |
