# Supplementary material for: Immunotherapy combined with apatinib in the treatment of advanced or metastatic gastric/gastroesophageal tumors: a systematic review and meta-analysis
Source: BMC Cancer. 2024 May 17;24:603. doi: 10.1186/s12885-024-12340-4 (PMC11102247; doi:10.1186/s12885-024-12340-4)
Supplement: Supplementary file 2 — Supplementary Material 2 [file 12885_2024_12340_MOESM2_ESM.docx]

Pubmed, Embase ,Web of Scienc and Cochrane Library all use the following search methods:（Immunotherapy or Immunotherapies or pembrolizumab OR toripalimab OR avelumab OR ipimumab OR atezolizumab OR nivolumab OR PD-1 OR PD-L1）AND（VEGFR-2 or apatinib or Antiangiogenic Drug or rivoceranib mesylate or YN968D1 or YN-968D1 or rivoceranib or apatinib mesylate）AND (gastric cancer or gastroesophageal junction or Esophagogastric junction or Stomach Neoplasm or Gastric Neoplasm)
