# Supplementary figures and images for: Immunotherapy combined with apatinib in the treatment of advanced or metastatic gastric/gastroesophageal tumors: a systematic review and meta-analysis
Source: BMC Cancer. 2024 May 17;24:603. doi: 10.1186/s12885-024-12340-4 (PMC11102247; doi:10.1186/s12885-024-12340-4)

Supplementary Figure 1: Funnel plot:(A): CR ; (B) : ORR(IA) ; (C) : ORR(IAC) ; (D) DCR(IA) ; (E) : DCR(IAC)


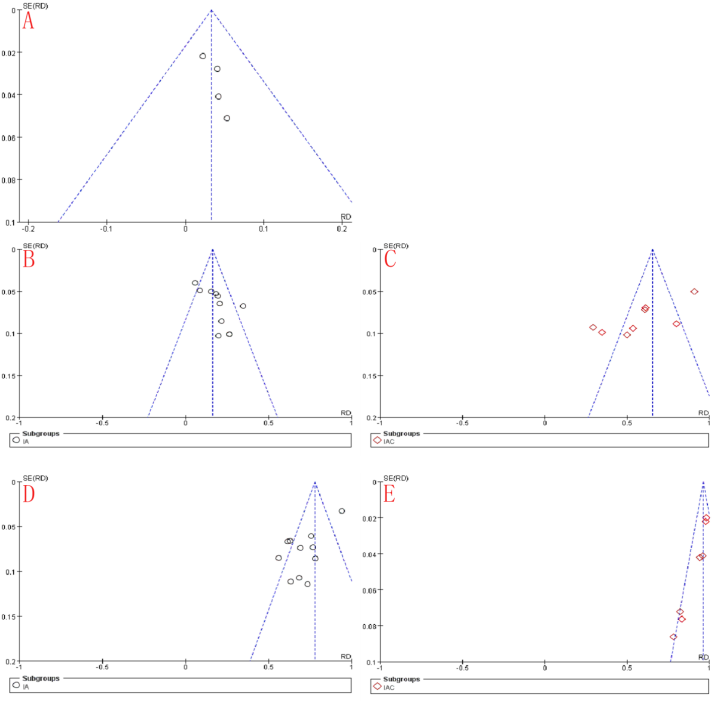

Supplement: Supplementary file 3 — Supplementary Material 3 [file 12885_2024_12340_MOESM3_ESM.docx]
